# Supplementary material for: Essential strategies for the detection of constitutive and ligand-dependent Gi-directed activity of 7TM receptors using bioluminescence resonance energy transfer
Source: bioRxiv. 2024 Dec 9:2024.12.04.626681. Preprint. [Version 1] doi: 10.1101/2024.12.04.626681 (PMC11661105; doi:10.1101/2024.12.04.626681)
Supplement: Supplement 1 [file NIHPP2024.12.04.626681v1-supplement-1.pdf]

bioRxiv preprint doi: https://doi.org/10.1101/2024.12.04.626681; this version posted December 9, 2024. The copyright holder for this preprint (which was not certified by peer review) is the author/funder, who has granted bioRxiv a license to display the preprint in perpetuity. It is made available under aCC-BY 4.0 International license.

Supplemental Figures and Legends

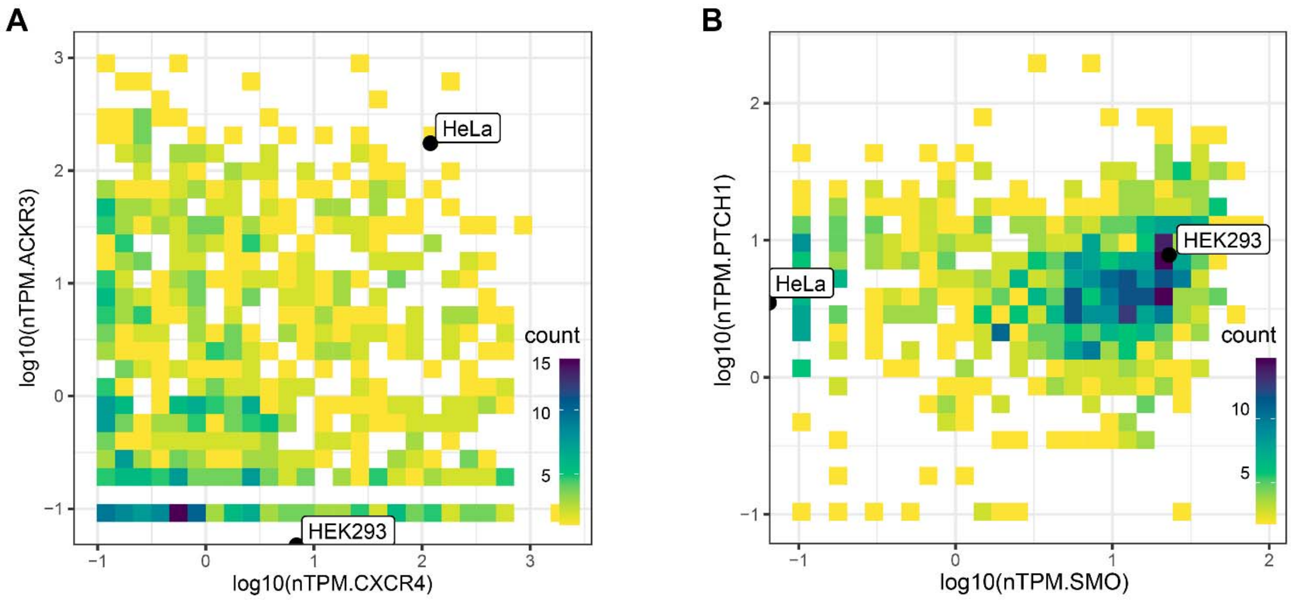

Supplementary Figure 1. Endogenous co-expression of receptors in this study in various cell lines.

(**A, B**) Normalized mRNA expression (in Transcripts Per Million, nTPM) of CXCR4 vs ACKR3 (**A**) and SMO vs PTCH1 (**B**) in a panel of 1055 cell lines from the Human Protein Atlas [86]. HeLa and HEK293T cells used in the study are labeled.

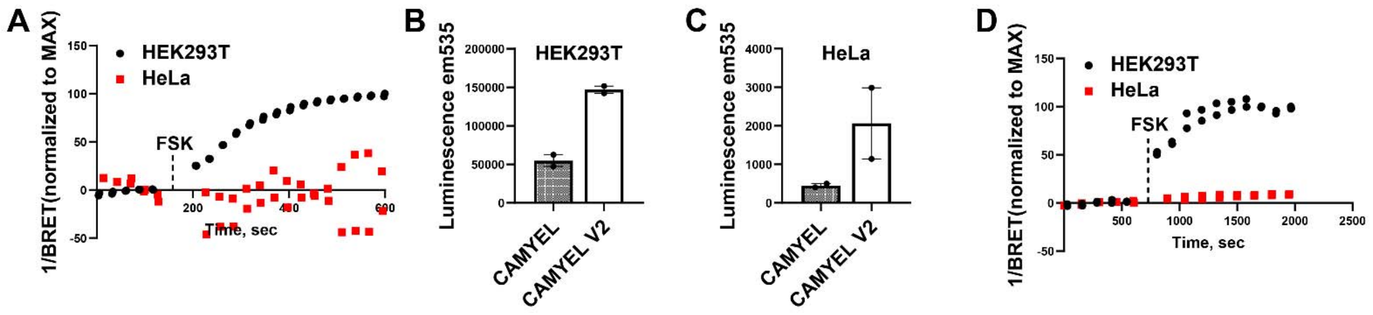

Supplementary Figure 2. CAMYEL vs CAMYEL-V2 biosensor.

(**A**) Mapping the time course of changes in CAMYEL 1/BRET ratios in HEK293T or HeLa cells upon the addition of Forskolin. (**B, C**) Absolute emission in the 505-590 nm range for the CAMYEL vs CAMYEL-V2 biosensor in HEK293T (**B**) and HeLa (**C**) cells, measured at 5-8 min post CTZ-h addition. (**D**) Time course of changes in CAMYEL-V2 1/BRET ratios in HEK293T or HeLa cells upon the addition of Forskolin.

bioRxiv preprint doi: https://doi.org/10.1101/2024.12.04.626681; this version posted December 9, 2024. The copyright holder for this preprint (which was not certified by peer review) is the author/funder, who has granted bioRxiv a license to display the preprint in perpetuity. It is made available under aCC-BY 4.0 International license.

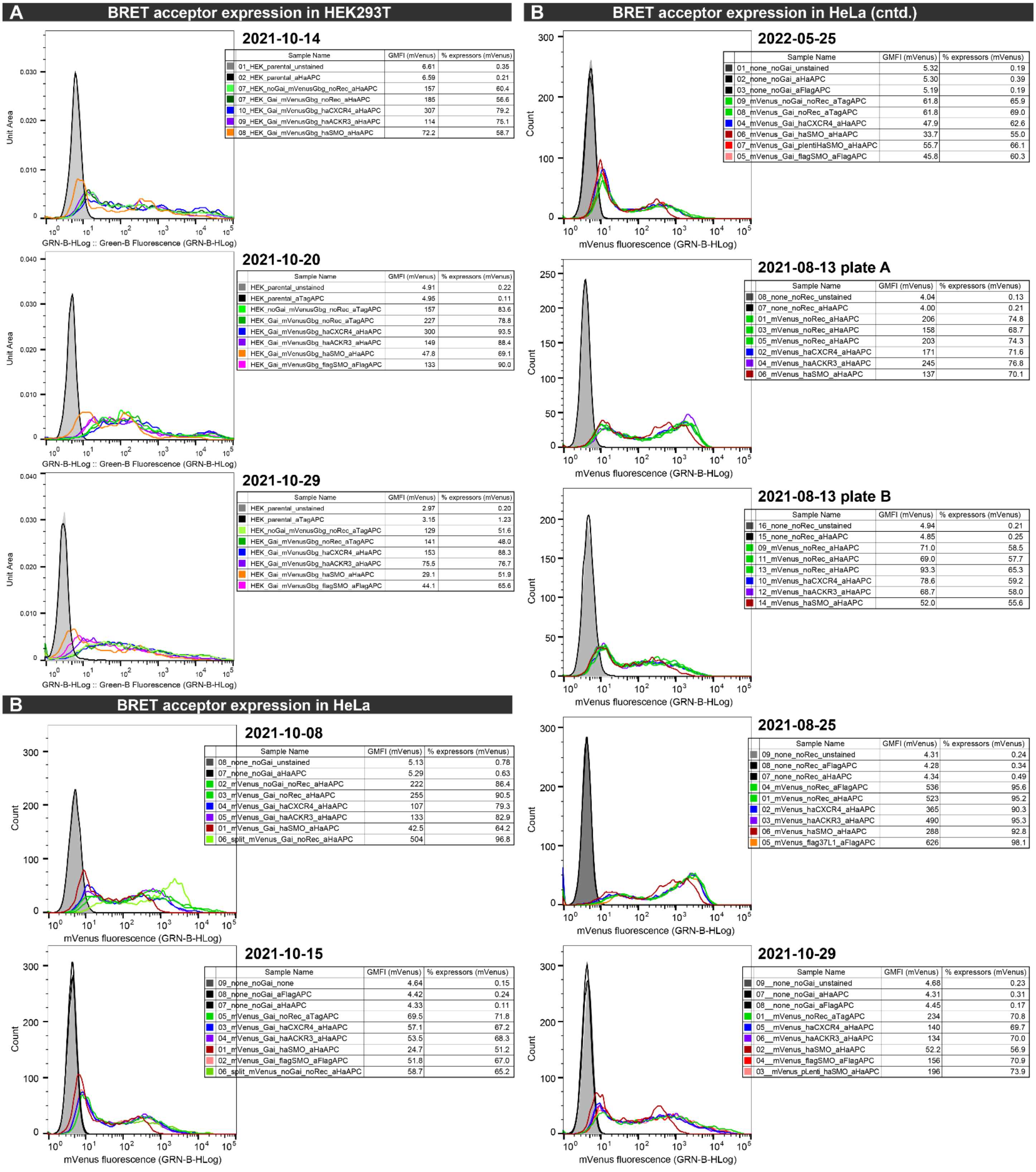

A — BRET acceptor expression in HEK293T

2021-10-14

Sample Name	GMFI (mVenus)	% expressors (mVenus)
01_HEK_parental_unstained	6.61	0.35
02_HEK_parental_aHaAPC	6.59	0.21
07_HEK_noGai_mVenusGbg_noRec_aHaAPC	157	60.4
07_HEK_Gai_mVenusGbg_noRec_aHaAPC	185	56.6
10_HEK_Gai_mVenusGbg_haCXCR4_aHaAPC	307	79.2
09_HEK_Gai_mVenusGbg_haACKR3_aHaAPC	114	75.1
08_HEK_Gai_mVenusGbg_haSMO_aHaAPC	72.2	58.7

2021-10-20

Sample Name	GMFI (mVenus)	% expressors (mVenus)
HEK_parental_unstained	4.91	0.22
HEK_parental_aTagAPC	4.95	0.11
HEK_noGai_mVenusGbg_noRec_aTagAPC	157	83.6
HEK_Gai_mVenusGbg_noRec_aTagAPC	227	78.8
HEK_Gai_mVenusGbg_haCXCR4_aHaAPC	300	93.5
HEK_Gai_mVenusGbg_haACKR3_aHaAPC	149	88.4
HEK_Gai_mVenusGbg_haSMO_aHaAPC	47.8	69.1
HEK_Gai_mVenusGbg_flagSMO_aFlagAPC	133	90.0

2021-10-29

Sample Name	GMFI (mVenus)	% expressors (mVenus)
HEK_parental_unstained	2.97	0.20
HEK_parental_aTagAPC	3.15	1.23
HEK_noGai_mVenusGbg_noRec_aTagAPC	129	51.6
HEK_Gai_mVenusGbg_noRec_aTagAPC	141	48.0
HEK_Gai_mVenusGbg_haCXCR4_aHaAPC	153	88.3
HEK_Gai_mVenusGbg_haACKR3_aHaAPC	75.5	76.7
HEK_Gai_mVenusGbg_haSMO_aHaAPC	29.1	51.9
HEK_Gai_mVenusGbg_flagSMO_aFlagAPC	44.1	65.6

B — BRET acceptor expression in HeLa

2021-10-08

Sample Name	GMFI (mVenus)	% expressors (mVenus)
08_none_noGai_unstained	5.13	0.78
07_none_noGai_aHaAPC	5.29	0.63
02_mVenus_noGai_noRec_aHaAPC	222	86.4
03_mVenus_Gai_noRec_aHaAPC	255	90.5
04_mVenus_Gai_haCXCR4_aHaAPC	107	79.3
05_mVenus_Gai_haACKR3_aHaAPC	133	82.9
01_mVenus_Gai_haSMO_aHaAPC	42.5	64.2
06_split_mVenus_Gai_noRec_aHaAPC	504	96.8

2021-10-15

Sample Name	GMFI (mVenus)	% expressors (mVenus)
09_none_noGai_none	4.64	0.15
08_none_noGai_aFlagAPC	4.42	0.24
07_none_noGai_aHaAPC	4.33	0.11
05_mVenus_Gai_noRec_aTagAPC	69.5	71.8
03_mVenus_Gai_haCXCR4_aHaAPC	57.1	67.2
04_mVenus_Gai_haACKR3_aHaAPC	53.5	68.3
01_mVenus_Gai_haSMO_aHaAPC	24.7	51.2
02_mVenus_Gai_flagSMO_aFlagAPC	51.8	67.0
06_split_mVenus_noGai_noRec_aHaAPC	58.7	65.2

B — BRET acceptor expression in HeLa (cntd.)

2022-05-25

Sample Name	GMFI (mVenus)	% expressors (mVenus)
01_none_noGai_unstained	5.32	0.19
02_none_noGai_aHaAPC	5.30	0.39
03_none_noGai_aFlagAPC	5.19	0.19
09_mVenus_noGai_noRec_aTagAPC	61.8	65.9
08_mVenus_Gai_noRec_aTagAPC	61.8	69.0
04_mVenus_Gai_haCXCR4_aHaAPC	47.9	62.6
06_mVenus_Gai_haSMO_aHaAPC	33.7	55.0
07_mVenus_Gai_plentiHaSMO_aHaAPC	55.7	66.1
05_mVenus_Gai_flagSMO_aFlagAPC	45.8	60.3

2021-08-13 plate A

Sample Name	GMFI (mVenus)	% expressors (mVenus)
08_none_noRec_unstained	4.04	0.13
07_none_noRec_aHaAPC	4.00	0.21
01_mVenus_noRec_aHaAPC	206	74.8
03_mVenus_noRec_aHaAPC	158	68.7
05_mVenus_noRec_aHaAPC	203	74.3
02_mVenus_haCXCR4_aHaAPC	171	71.6
04_mVenus_haACKR3_aHaAPC	245	76.8
06_mVenus_haSMO_aHaAPC	137	70.1

2021-08-13 plate B

Sample Name	GMFI (mVenus)	% expressors (mVenus)
16_none_noRec_unstained	4.94	0.21
15_none_noRec_aHaAPC	4.85	0.25
09_mVenus_noRec_aHaAPC	71.0	58.5
11_mVenus_noRec_aHaAPC	69.0	57.7
13_mVenus_noRec_aHaAPC	93.3	65.3
10_mVenus_haCXCR4_aHaAPC	78.6	59.2
12_mVenus_haACKR3_aHaAPC	68.7	58.0
14_mVenus_haSMO_aHaAPC	52.0	55.6

2021-08-25

Sample Name	GMFI (mVenus)	% expressors (mVenus)
09_none_noRec_unstained	4.31	0.24
08_none_noRec_aFlagAPC	4.28	0.34
07_none_noRec_aHaAPC	4.34	0.49
04_mVenus_noRec_aFlagAPC	536	95.6
01_mVenus_noRec_aHaAPC	523	95.2
02_mVenus_haCXCR4_aHaAPC	365	90.3
03_mVenus_haACKR3_aHaAPC	490	95.3
06_mVenus_haSMO_aHaAPC	288	92.8
05_mVenus_flag37L1_aFlagAPC	626	98.1

2021-10-29

Sample Name	GMFI (mVenus)	% expressors (mVenus)
09__none_noGai_unstained	4.68	0.23
07__none_noGai_aHaAPC	4.31	0.31
08__none_noGai_aFlagAPC	4.45	0.17
01__mVenus_noRec_aTagAPC	234	70.8
05__mVenus_haCXCR4_aHaAPC	140	69.7
06__mVenus_haACKR3_aHaAPC	134	70.0
02__mVenus_haSMO_aHaAPC	52.2	56.9
04__mVenus_flagSMO_aFlagAPC	156	70.9
03__mVenus_pLenti_haSMO_aHaAPC	196	73.9

Supplementary Figure 3. Quantification of BRET acceptor expression by flow cytometry.
Flow cytometry assessment of BRET acceptor expression in samples co-transfected or not with the indicated receptors, in individual biological replicates of BRET experiments presented in the paper for HEK293T cells (**A**) and HeLa cells (**B**).

bioRxiv preprint doi: https://doi.org/10.1101/2024.12.04.626681; this version posted December 9, 2024. The copyright holder for this preprint (which was not certified by peer review) is the author/funder, who has granted bioRxiv a license to display the preprint in perpetuity. It is made available under aCC-BY 4.0 International license.

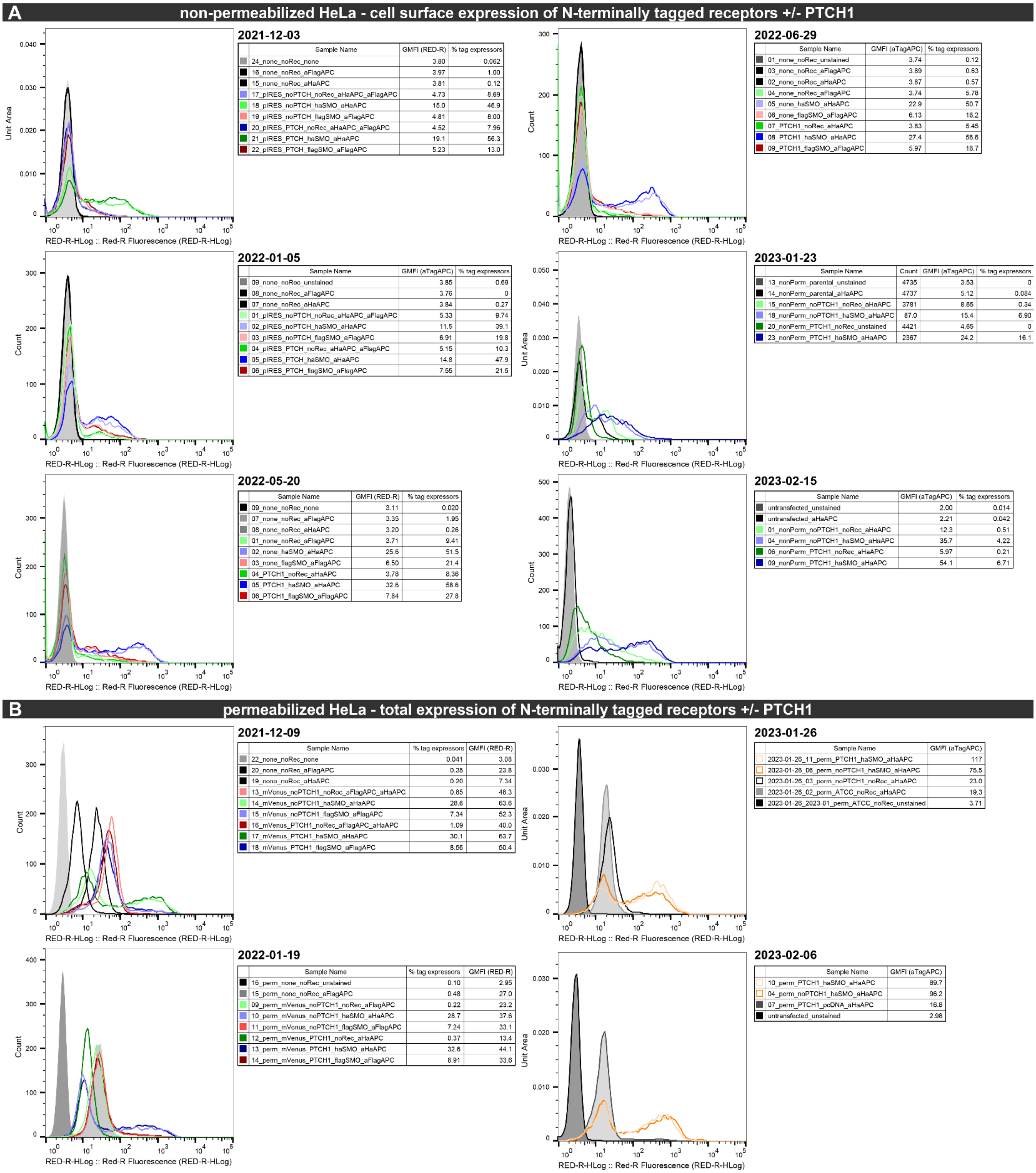

Supplementary Figure 4. Quantification of SMO expression in the presence or absence of PTCH1 by flow cytometry.

Flow cytometry quantification of surface (**A**) or total (**B**) expression of indicated SMO constructs in HeLa cells in independent biological experiments. N-terminally HA- or Flag-tagged receptors were detected using an APC-conjugated anti-HA antibody.

bioRxiv preprint doi: https://doi.org/10.1101/2024.12.04.626681; this version posted December 9, 2024. The copyright holder for this preprint (which was not certified by peer review) is the author/funder, who has granted bioRxiv a license to display the preprint in perpetuity. It is made available under aCC-BY 4.0 International license.

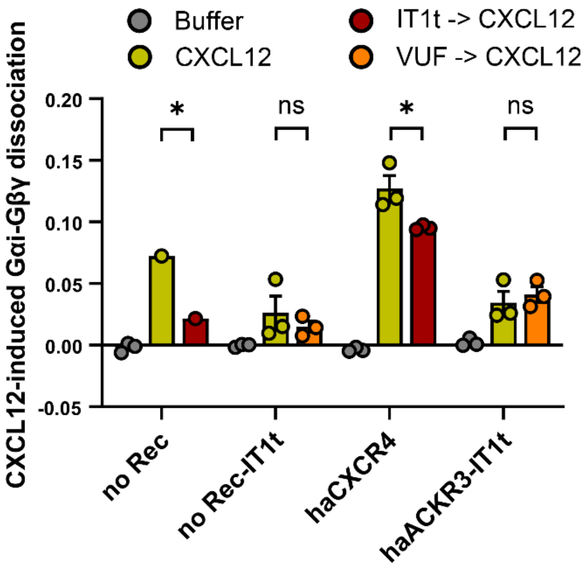

Supplementary Figure 5. CXCL12-induced changes in Gαi-Gβγ dissociation.

CXCL12-induced changes in Gαi-Gβγ dissociation in HEK293T cells transfected with the indicated receptors pre-treated or not with 500nM IT1t (a CXCR4 antagonist) or VUF16840 (an ACKR3 antagonist).
